# Tailoring the crystal growth of quartz on silicon for patterning epitaxial piezoelectric films†

**DOI:** 10.1039/c9na00388f

**Published:** 2019-08-29

**Authors:** Qianzhe Zhang, David Sánchez-Fuentes, Andrés Gómez, Rudy Desgarceaux, Benoit Charlot, Jaume Gàzquez, Adrián Carretero-Genevrier, Martí Gich

**Affiliations:** Institut de Ciència de Materials de Barcelona (ICMAB), Consejo Superior de Investigaciones Científicas (CSIC) Campus UAB 08193 Bellaterra Catalonia Spain marti.gich@csic.es; Institut d'Électronique et des Systèmes (IES), UMR 5214, CNRS, Université de Montpellier 860 rue Saint Priest 34095 Montpellier France carretero@ies.univ-montp2.fr

## Abstract

Epitaxial films of piezoelectric α-quartz could enable the fabrication of sensors with unprecedented sensitivity for prospective applications in electronics, biology and medicine. However, the prerequisites are harnessing the crystallization of epitaxial α-quartz and tailoring suitable film microstructures for nanostructuration. Here, we bring new insights into the crystallization of epitaxial α-quartz films on silicon (100) from the devitrification of porous silica and the control of the film microstructures: we show that by increasing the quantity of devitrifying agent (Sr) it is possible to switch from an α-quartz microstructure consisting of a porous flat film to one dominated by larger, fully dense α-quartz crystals. We also found that the film thickness, relative humidity and the nature of the surfactant play an important role in the control of the microstructure and homogeneity of the films. *Via* a multi-layer deposition method, we have extended the maximum thickness of the α-quartz films from a few hundreds of nm to the μm range. Moreover, we found a convenient method to combine this multilayer approach with soft lithography to pattern silica films while preserving epitaxial crystallization. This improved control over crystallization and the possibility of preparing patterned films of epitaxial α-quartz on Si substrates pave the path to future developments in applications based on electromechanics, optics and optomechanics.

## Abstract 2

Epitaxial films of piezoelectric α-quartz could enable the fabrication of sensors with unprecedented sensitivity for prospective applications in electronics, biology and medicine.

## Graphical abstract

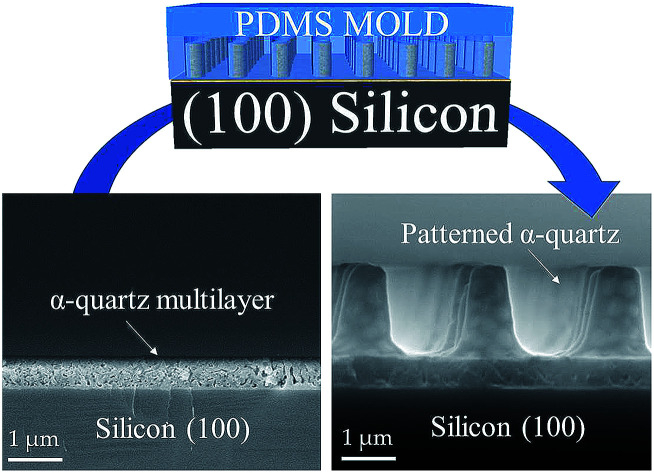

## 1. Introduction

Silicon and oxygen are the most abundant elements in the earth's crust. Accordingly, silica (SiO_2_), in the form of [SiO_4_] tetrahedral networks, is a basic building block of most minerals. For centuries, those minerals have been massively exploited for the production of building materials such as glass or concrete, but more recently silicates have found a wide range of high-end applications, from the use of zeolites in catalysis to the incorporation of lutetium silicates in scintillators.^1^ Quartz single crystals are a remarkable example of such high technology applications of minerals. Thanks to their piezoelectric properties with a high resonance quality factor, thin quartz plates are used as electric oscillators providing a stable clock frequency in virtually any electronic appliance. Since the resonant frequency of the plates is extremely sensitive to their mass and thickness, quartz crystals are also exploited in microgravimetric devices for sensing applications.^2,3^ Moreover, thanks to their non-centrosymmetric and chiral structure, quartz crystals present optical activity, finding applications in optics.^4^

Fully exploiting the potential of quartz and other functional silicates in current and future applications requires controlling their microstructure down to the nanoscale. Yet, despite the importance of silicates in leading edge applications, fundamental aspects of their crystallisation are still poorly understood. A paradigmatic example of this is the highly empirical and poorly predictive devitrification of amorphous silicates. Indeed, even controlling the crystallization of SiO_2_ is a long-standing problem related to the remarkable stability of silica and the tiny differences in free energies of formation between all its amorphous and crystalline forms.^5^

Lately, fundamental and application-driven interest in nanoscale SiO_2_ provided new insights towards understanding and controlling silica devitrification in microporous, mesoporous and ultrathin silica.^6–15^ Most of these studies highlight the essential role of alkaline,^11^ alkaline earth^8–10,15^ and transition metal dopants^7^ in obtaining the desired crystallization. For instance, the formation of iron silicate seeds underpins the epitaxial growth of thermally evaporated ultrathin silica on Ru (0001).^13^ Other studies show that, at the nanoscale, the confinement of alkaline or alkaline earth melting agents is essential for achieving a controlled crystallization of α-quartz. In particular, we prepared superlattices of polycrystalline α-quartz nanospheres by impregnating preformed 3D stackings of mesoporous silica spheres with aqueous solutions of alkaline earth cations followed by thermal treatments above 800 °C. However, using a Li^3+^ solution we observed a straightforward crystallization of α-quartz and lithium silicate with a loss of the superstructure morphology, at temperatures as low as 650 °C. In contrast, Matsuno *et al.*^11^ obtained α-quartz nanosphere superlattices with improved crystallinity at 870 °C after confining Li^+^ between silica spheres and a reinforcing carbon coating. Ndayishimiye *et al.*^12^ impregnated microporous SiO_2_ nanoparticles with NaOH solutions and studied their hydrothermal sintering, finding an amorphous product below a concentration threshold (1 M) while dense polycrystalline α-quartz resulted from impregnations with more concentrated solutions.^12^

Also, the use of surfactants in the synthesis of mesoporous silica can strongly influence the devitrification. For instance, with the presence of amphiphiles the temperature of silica crystallization under high pressure was lower than in the case where the surfactants had been previously removed.^14^ In this sense, Putz *et al.* used sodium metasilicate solutions in microemulsions to obtain small α-quartz nanocrystals at room temperature or under mild hydrothermal conditions below 200 °C.^15^ The above examples illustrate the complexity of controlling the crystallization and microstructure of nanostructured α-quartz and highlight the critical roles of the silica porosity, the concentration, distribution and confinement of devitrifying agents or the presence of surfactants.

We recently developed a chemical solution deposition method for preparing epitaxial α-quartz films on Si (100) substrates. The method relies on the thermal devitrification of dip-coated mesoporous silica films, assisted by alkaline earth cations in amphiphilic molecular templates.^8^ By varying the surfactants and the synthesis conditions the porosity of the mesoporous silica can be tailored to range from a few tens of nm to 1 μm and is preserved upon crystallization. But pore sizes below 40 nm cannot be retained in the crystalline replica: the films display rough surfaces of densely packed crystals with lateral sizes below 500 nm coexisting with significantly larger single crystalline domains attaining several tens of μm. Such single crystalline domains of fully dense α-quartz can be appealing for fabricating nanostructured optical metamaterials and devices,^16^ minimizing the scattering losses. In contrast, for microgravimetric sensors, it would be advantageous to have films with low roughness (enabling the fabrication of surface acoustic wave devices by lithography) and some degree of porosity (to achieve an increased mass sensitivity).

A prerequisite to enable these technological applications is gaining a greater control over the crystallization of epitaxial quartz. On the one hand, a method to prepare porous films displaying low roughness has not been reported so far. On the other hand, the synthesis of large single crystalline dense α-quartz follows a quite inconvenient 2-step process.^8^ This involves the impregnation of a preformed silica film in a solution of the devitrifying agent, and the need for cleaning the film surface from an excess of impregnating solution entails irreproducibility issues. Moreover, most applications require controlling the film thickness from hundreds of nm to above 1 μm, but the maximum thicknesses of epitaxial quartz films prepared by the dip coating technique do not exceed a few hundred nm.

To address these shortcomings our aim was to control the microstructure of epitaxial α-quartz films on silicon. We focused on understanding the roles of the concentration of the devitrifying agent (Sr^2+^), the relative humidity and the film roughness and thickness, which has been poorly studied so far. These proved to be crucial for tailoring the film microstructure and implementing a procedure to increase the film thickness up to the micron range. We exploited this enhanced control over the crystallization to prepare high-quality patterns of epitaxial quartz films by soft lithography.

## 2. Experimental

### 2.1 Synthesis

#### 2.1.1 Solution preparation

All the chemicals were from Sigma-Aldrich and without any further purification. In a typical process, we first prepared Solution A by adding 0.7 g Brij-58 into 23.26 g absolute ethanol, followed by adding 1.5 g HCl (37%) and 4.22 g tetraethyl orthosilicate (TEOS) and then the solution was stirred for at least 4 h and not more than 18 h. After that, an aqueous solution of Sr^2+^ was prepared with SrCl_2_ (Solution B). The solution used to prepare Sr-doped mesoporous silica films by dip-coating (Solution C) was obtained by adding 275 μL Solution B into 10 mL of as-prepared Solution A and stirring it for 10 min. The films were always obtained no later than 40 min after preparing Solution C, as Sr^2+^ is not stable in the latter. To study the effect of changing the Sr content in the films, we varied the molarity of Solution B between 0.4 M and 2 M. By mixing fixed volumes of Solution A and Solution B we could prepare films with different Sr contents without changing the water content in Solution C. Control films were also prepared without the addition of SrCl_2_. As a reference, for 1 M Solution B, the molar composition of Solution C was TEOS : Brij-58 : HCl : EtOH : SrCl_2_ = 1 : 0.3 : 0.7 : 25 : 0.05 having a Sr/SiO_2_ molar ratio of 0.05. For samples with other Sr contents, the molar ratio of SrCl_2_ with respect to TEOS is given by 0.05 times the corresponding molarity of Solution B. Taking this into account the SrCl_2_/TEOS molar ratios studied in this work (and the corresponding Sr/SiO_2_ molar ratios of the quartz films) range from 0.02 to 0.1 (see Table S1†). Thus, hereafter the Sr content of the different quartz films is expressed by the Sr/SiO_2_ molar ratios, labeled *R*_Sr_. To explore the effect of different types of surfactants we prepared films in which the oligomeric alkyl poly(ethylene oxide) Brij-58 was replaced by alternative surfactants such as the ionic cetyltrimethylammonium bromide (CTAB) and the poly(ethylene oxide) triblock copolymer Pluronic F127, with the following reference compositions for Solution C TEOS : CTAB : HCl : EtOH : SrCl_2_ = 1 : 0.14 : 0.7 : 25 : 0.05 and TEOS : F127 : HCl : EtOH : SrCl_2_ = 1 : 0.0055 : 0.7 : 25 : 0.05.

#### 2.1.2 Gel films by dip-coating

Mono-layer gel films on 525 μm thick boron-doped Si (100) substrates with a conductivity of 5–10 ohm cm (Active Business Company GmbH) and typical dimensions of 2 cm by 5 cm were prepared with a ND-DC300 dip-coater (Nadetech Innovations) equipped with an EBC10 Miniclima Device to control the surrounding temperature and relative humidity. During the dip-coating, we fixed the ambient temperature and relative humidity as 25 °C and 40% and the thickness of the film was controlled by the withdrawal rate. In this study, all the films were made at a withdrawal rate of 5 mm s^−1^ (the maximum allowed by the equipment) except those intended for the study of the thickness control. After dip-coating, the as-prepared gel films were consolidated with a thermal treatment of 5 min at 450 °C under an air atmosphere. The multi-layer gel films were obtained by repeating the required number of times the process of mono-layer preparation on the same substrate. After the gel consolidation the bottom ends of the substrates containing the meniscus and characterized by a non-uniform thickness were cut-out, as explained elsewhere.^17^

#### 2.1.3 Crystallization

The as-prepared gel films were introduced into a furnace already at 1000 °C in an air atmosphere and held at this temperature for 300 min, except for those films intended to study the influence of crystallization time. The crystallized films were recovered after natural cooling of the furnace to room temperature.

### 2.2 Nanostructuration process

#### 2.2.1 Preparation of molds

Silicon masters were fabricated with columns reaching 2 μm in height and 1 μm in diameter using LIL lithography. For that, we used a positive photoresist, AZ MIR 701, which was exposed using the interferential lithography technique to obtain a network of dots after using a developer, AZ726. This procedure allows us to rapidly obtain periodic designs over a large surface (∼cm^2^) without the need for a lithographic mask.^18^ To produce an interferometric pattern with a pitch of 1 μm, a 405 nm wavelength laser with a divergent beam was reflected by two mirrors shifted with an angle of 10°. Then, the dot patterns were obtained by two exposures. The first exposure created periodic lines and a second exposure, shifted by 90° with respect to the first exposure, generated perpendicular periodic lines. The result of these two exposures, after development, generates the dots. The silicon was anisotropically etched by inductively coupled plasma reactive ion etching (ICP-RIE) (model corial 200 IL) using the CHF_3_/O_2_ gas mixture. RIE conditions for etching and producing a periodic pattern of silicon pillars of 1 μm depth were the following: power: 120 W RF, 400 W LF, gas: CHF_3_ 100 sccm–O_2_ 20 sccm (standard cubic centimeter per minutes), pressure: 10 mTorr and time: 10 min. ICP-RIE produces a dry and directional etching induced by a mixture of CHF_3_ and O_2_ plasma.

#### 2.2.2 Preparation of polydimethylsiloxane (PDMS) molds and the printing process

PDMS reactants (90 wt% RTV141A; 10 wt% RTV141B from BLUESIL) were transferred onto the master and dried at 70 °C for 1 h before unmolding. Then, four amorphous silica layers were deposited sequentially at a constant relative humidity of 45% with controlled withdrawal speeds of 300 mm min^−1^ in order to adjust the final quartz thickness to 600 nm, being successively consolidated at 450 °C for 10 min. After the last dip-coating, the substrates were quickly introduced for 1 min into a custom-designed chamber at a controlled temperature of 25 °C at a constant humidity of 45%. Imprinting of sol–gel films with a PDMS mold involves the following steps. First, molds were degassed under vacuum (10 mbar) for 20 min before direct application on the as-prepared xerogel films kept in a controlled environment, without additional pressure. After 1 min, the samples were transferred to a 70 °C stove for 2 min and then to a 120 °C stove for 10 min to consolidate the xerogel films before manually peeling off the PDMS mold. Next, the sol–gel replicas were annealed at 450 °C for 10 min for consolidation. Finally, the sample was crystallized at 1000 °C for 5 h in an air atmosphere.

### 2.3 Structural characterization and piezoelectric measurements

#### 2.3.1 X-ray diffraction (XRD)

The crystalline textures and rocking curve measurements of the films were performed on a Bruker D8 diffractometer (3 s acquisition every 0.02° in Bragg–Brentano geometry, with a radiation wavelength of 0.154056 nm). The epitaxial relationship was analyzed through X-ray diffraction measurements by using a Bruker AXS GADDS equipped with a 2D X-ray detector. The XRD maps were obtained on a Bruker D8 discovery equipped with a 2D detector.

#### 2.3.2 Optical microscope

Optical images of the films were obtained using an Olympus BX51M optical microscope equipped with a Nikon DS-Fi3 camera.

#### 2.3.3 Field emission gun scanning electron microscopy (FEG-SEM)

The microstructures of the films were investigated with a FEG-SEM model Su-70 Hitachi, equipped with an EDX detector X-max 50 mm^2^ from Oxford instruments. The analysis of the images to obtain the area coverage by the different microstructures was done with the ImageJ software.^19^ For this, the whole picture area was measured and the images were transformed into 16-bit black-and-white images. Then the contrast was adjusted to make the different microstructures more distinguishable. A luminosity threshold was set for the software to automatically select the zones with dark contrast and provide their areas.

#### 2.3.4 Transmission electron microscopy (TEM)

Cross-sectional studies of the films were performed by using an FEI Titan3 operated at 80 kV and equipped with a superTwin® objective lens and a CETCOR Cs-objective corrector from CEOS Company.

#### 2.3.5 Atomic force microscopy (AFM)

The topography of quartz films was studied using tapping Atomic Force Microscopy (AFM) images obtained using a Park Systems NX-10 Scanning Probe Microscopy (SPM) unit. Piezoelectric characterization through the direct piezoelectric effect was made by Direct Piezoelectric Force Microscopy^20^ using an Agilent 5500LS instrument equipped with a low leakage amplifier (Analog Devices ADA4530) with solid Pt tips (Rockymountain Nanotechnology RMN-25 PtIr200H). A Periodically Poled Lithium Niobate from the Bruker AFM was used as a reference testing platform. The roughness of the films was obtained from a 50 μm × 50 μm AFM image by using the Gwyddion software.^21^

#### 2.3.6 X-ray photoelectron spectroscopy (XPS)

The measurement was taken *via* a SPECS PHOIBOS 150 hemispherical energy analyser and analysis was taken with the help of the CasaXPS software.

## 3. Results and discussion

### 3.1 Influence of the Sr content in the microstructure of epitaxial quartz

**Fig. 1:**
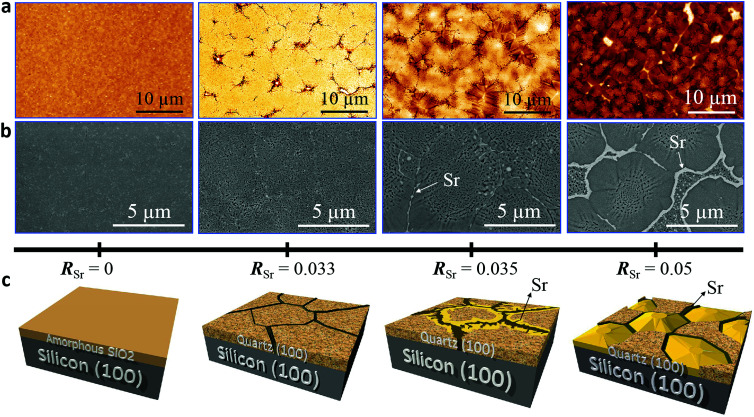
The microstructures of α-quartz films containing different amounts of Sr (0 < *R*_Sr_ < 0.05 indicated in a scale below the images) are revealed by AFM (a) and SEM in backscattered electron mode (b). The salient features of these microstructures are illustrated in the 3D model (c). Those films were prepared with a withdrawal speed of 5 mm s^−1^ under a relative humidity of 40% at 25 °C using Brij-58 as the surfactant. The annealing treatment was at 1000 °C for 300 minutes in an air atmosphere.

We performed a systematic study on the influence of Sr content in the microstructure, crystallinity and epitaxial growth quality of quartz films obtained following the same annealing treatment at 1000 °C. For this, we prepared porous silica films under identical deposition conditions (see Gel films by dip coating in Section 2.1) with Sr/SiO_2_ molar ratios (*R*_Sr_) between 0 and 0.1. Fig. 1 shows the evolution of the characteristic microstructures of the film surfaces upon increasing the Sr content as observed from AFM topography images (Fig. 1a) and SEM in backscattered electron mode (Fig. 1b).

Fig. 1c provides a sketch emphasizing the salient features of the microstructural changes induced by increasing the Sr content, which we describe below. The films without Sr, (leftmost images in Fig. 1a and b), are characterized by their homogeneity with some cracks and sparse pores (see Fig. S1†). In contrast, the Sr-containing films present crevices which define roughly elliptical domains of sizes between 5 and 10 μm in an arrangement reminiscent of mud-cracking patterns, but with the presence within the domains of many pores of sizes below 300 nm. Notice that for the films with *R*_Sr_ > 0.033 (two rightmost images in Fig. 1a and b), in the areas bordering the domain boundaries, the porosity has given way to fully dense faceted structures which are characteristic of crystals. These dense structures (the yellow zone in Fig. 1c) become more prominent as *R*_Sr_ increases and prevail for *R*_Sr_ = 0.05, leaving only a residual porous zone at the center of the domains. The SEM images (Fig. 1b) show that these dense structures are accompanied by bright white contrasts at the domain boundaries which were ascribed to accumulations of Sr, the presence of which was confirmed by XPS (see Fig. S2†). Note that these Sr-rich areas present irregular and droplet-like shapes in the regions with porous microstructures, suggesting that these have been a liquid phase at the annealing temperature. However, at some point during the cooling the Sr-rich phase undergoes a process of carbonation and SrCO_3_ is its major component at room temperature, as we previously reported.^9^ The AFM images of the films with dense structures (see Fig. 1a) present a more rugged topography. This is reflected in the fivefold increase of the root mean square roughness (from 5 nm to 25 nm) for *R*_Sr_ increasing from 0.033 to 0.1 (see Fig. S3†).

**Fig. 2:**
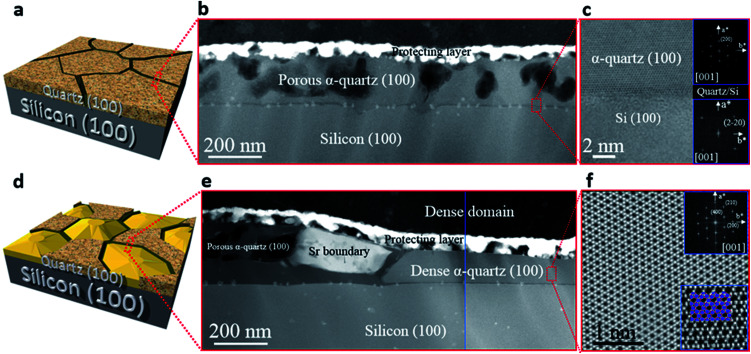
Cross-sectional STEM images: (a) and (d) are the 3D representations for two different microstructures with the red dotted lines indicating the cross-sections to which the images correspond. (b) Low magnification *Z*-contrast image of the porous film (*R*_Sr_ = 0.033). (c) Bright field STEM atomic resolution image of the porous film cross-section showing the interface between the α-quartz (100) film and silicon (100) substrate. The insets are the FFT patterns of the film and substrate, respectively. (e) Low magnification *Z*-contrast image of the dense film (*R*_Sr_ = 0.05). (f) Bright field STEM atomic resolution image of the dense film cross-section. The upper inset is the FFT pattern and the lower inset a higher magnification image displaying the model structure with the tetrahedral Si in blue, surrounded by oxygens in red.

**Fig. 3:**
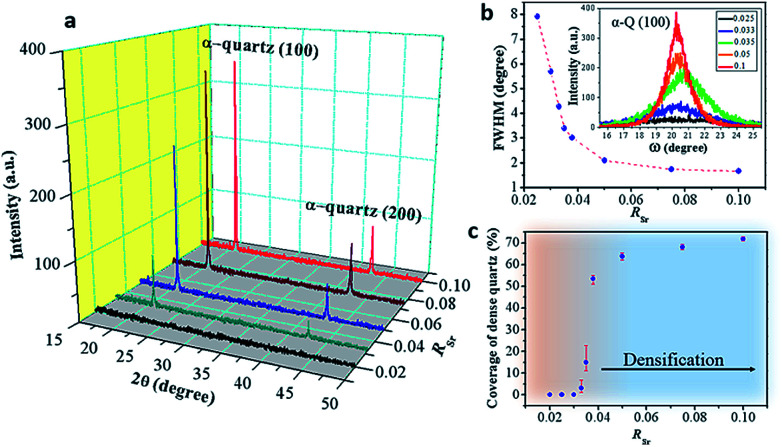
(a) XRD *θ*–2*θ* scan results of films with different *R*_Sr_. (b) Relationship between the FWHM of rocking curves of α-quartz (100) and *R*_Sr_. (c) Influence of *R*_Sr_ on the area coverage of dense α-quartz. Those films were prepared with a withdrawal speed of 5 mm s^−1^ under a relative humidity of 40% at 25 °C using Brij-58 as the surfactant. The annealing treatment was at 1000 °C for 300 minutes in an air atmosphere.

We investigated the characteristic microstructure of the films with *R*_Sr_ = 0.033 (porous) and *R*_Sr_ = 0.05 (dense domains) by scanning transmission electron microscopy (STEM). Fig. 2b and e present *Z*-contrast low magnification images of the quartz films from the zones indicated by red-dotted lines in the 3D sketches, which represent the salient characteristics of the porous and dense films (Fig. 2a and d, respectively). Fig. 2b shows interconnected pores with diameters in the range of 50–100 nm. Distributed within the pores there are small whitish features of about 10–20 nm which correspond to Sr agglomerates. An analogous microstructure is observed on the left-hand side of Fig. 2e, next to a large Sr aggregate which marks the transition to a thinner dense structure on the right-hand side of the image. The atomic resolution images of Fig. 2c and f and the corresponding fast Fourier transform (FFT) patterns (shown in the insets) indicate that both the porous and dense structures are α-quartz crystals with well-defined orientations. In particular, the FFTs of Fig. 2c, which correspond to the orientation of the Si substrate and α-quartz crystals along their [100] zone axes, are indicative of an epitaxial growth with Si (100)//α-quartz (100) and Si (010)//α-quartz (010), in agreement with our previous report.^8^ Such epitaxial relationships have been confirmed both for porous and dense quartz films by X-ray diffraction, with *θ*–2*θ* scans (Fig. 3a) and pole figures of the Si {111} and α-quartz {101} reflections (see Fig. S4†).

Fig. 3a shows that the intensities of the (100) and (200) reflections of α-quartz increase with *R*_Sr_. In particular, the film with *R*_Sr_ = 0.02 does not present any diffraction peak. However, by observing this film with an optical microscope we noticed the presence of some crystals (see Fig. S5a–c†), which were not found in films with lower *R*_Sr_. The optical microscopy observations also suggested the presence of a residual amorphous phase for films with 0.02 < *R*_Sr_ < 0.033 which was confirmed by electron backscatter diffraction (EBSD) observations for the film with *R*_Sr_ = 0.02 (see Fig. S5d–f†). For *R*_Sr_ = 0.033 the film is fully crystallized and presents the characteristic porosity that we described above, which is not observed for amorphous or poorly crystallized films. This porosity originates from the larger density of α-quartz (2.65 g cm^−3^) compared to that of amorphous SiO_2_ (2.20 g cm^−3^)^22^ with the resulting need to accommodate the volume shrinkage upon crystallization. The densification of SiO_2_ is also responsible for the formation of domains delimited by cracks which, as we shall discuss below, play an important role in the formation of dense crystal structures. The inset of Fig. 3b presents a series of *θ*–*ω* measurements of the (100) α-quartz reflection (rocking curves) for films with different Sr contents, showing that their full widths at half maximum (FWHM) are lowered by increasing *R*_Sr_. The FWHM decreases steeply when *R*_Sr_ rises from 0.025 up to 0.035 but much more slowly and tending to level off for higher Sr concentrations. This indicates that the effect of *R*_Sr_ on the misorientation of α-quartz crystals follows two differentiated regimes delimited by *R*_Sr_ between 0.033 and 0.035. This coincides with the threshold concentration range above which dense films can be stabilized over the porous morphologies, as is evidenced in the plot of surface coverage by dense crystals *vs. R*_Sr_ (Fig. 3b). An equivalent sharp transition is observed at similar Sr contents in the evolution of film roughness *vs. R*_Sr_ as shown in Fig. S3.† It is quite remarkable that the crystallization of films with porous morphology (*R*_Sr_ = 0.033) does not generate a marked increase in the surface roughness as occurs for the crystallization of films with dense morphologies (*R*_Sr_ = 0.05). Indeed, for porous films the roughness can be as low as 4.6 nm, only slightly above that of an amorphous film before crystallization (4 nm). The larger mosaicity of porous films results from slight misorientations of different crystals as they nucleate on the substrate wherever there is enough Sr to allow the devitrification. However, their growth is limited by the availability of Sr which forms small size aggregates. Hence, the different crystals join forming low energy domain boundaries and the film planarity is maintained through the porosity that accommodates the volume collapse. When SiO_2_ crystallizes, Sr is expulsed into the empty spaces that have been created, forming larger aggregates. The accretion of Sr becomes more important as *R*_Sr_ increases and, for *R*_Sr_ > 0.033, Sr eventually starts filling the crevices of the domain boundaries (see Fig. 1). At this stage, a recrystallization of α-quartz starts at the interface of the Sr accumulation with the domain boundary and progresses inward. This results in larger dense crystals with low mosaicity, leaving a residual core of the primary porous crystallization. This indicates that the crystallization of Sr-rich films is a two-step process.

We have confirmed this sequence of events in a time dependent study of the crystallization on those films with the minimum *R*_Sr_ which allows obtaining dense structures (*R*_Sr_ = 0.035). The relatively low Sr content ensures that the crystallization is sufficiently slow to allow monitoring it by quenching films at different annealing times. The SEM and AFM images of films quenched after annealing at 1000 °C for 40, 50 and 60 minutes are presented in Fig. S6 and S8a,† respectively. At 40 min we can see isolated or a few aggregated domains of the primary porous crystalline phase free of Sr (note the darker contrast of the backscattered electron image) surrounded by non-porous zones much richer in Sr. The image for 50 min displays a full coverage of the film surface by porous domains and the beginning of the recrystallization from limited portions of the domain boundaries in which Sr has accumulated. At 60 min we see a more advanced stage of the inward growth from larger portions of the domain boundaries. An analogous time dependent study on films with *R*_Sr_ = 0.05 also revealed a primary crystallization of the characteristic porous microstructure but due to their higher Sr content the recrystallization occurs well before the porous domains can cover the whole film surface (see Fig. S7 and S8b†). This shows that the microstructure of the films is influenced by the dynamics of the Sr-rich liquid phase, which can be controlled by controlling *R*_Sr_, its distribution and the details of the annealing process.

In our films, the primary crystallization of α-quartz from amorphous silica is made possible by mobile Sr^2+^ ions from the Sr-rich droplets which weaken Si–O bonds, increasing the flexibility of [SiO_4_] tetrahedral units and the migration of oxygen. This mechanism has been studied thoroughly in the recrystallization and densification of α-quartz previously amorphized by the implantation of alkaline and alkaline earth ions^23–26^ or in hydrothermal solution.^12^ In the case of recrystallization, our observations suggest that the role of the Sr-rich liquid is akin to that of the fluxes widely used in solid state chemistry to improve sintering or crystal growth. There are recent examples of solid–liquid crystallization by an Ostwald ripening mechanism assisted by a flux,^27–30^ which leads us to hypothesize that an analogous mechanism could be at play in epitaxial α-quartz films obtained from silica gels with *R*_Sr_ > 0.033.

### 3.2 The roles of thickness, relative humidity and type of surfactant

Regardless of the major role of *R*_Sr_ in controlling the microstructure of α-quartz films, our systematic study also encompassed the influence of other important parameters of the film growth by dip-coating such as the withdrawal speed, the relative humidity and the type of surfactant.

#### 3.2.1 Surfactant and relative humidity

Relative humidity and type of surfactant are two important parameters governing the structure of gel films and, accordingly, can influence the microstructure of crystallized films. Our previous studies have shown the key role of surfactant in distributing and stabilizing Sr inside the films.^8,9^ In the present study, most of the films were prepared with the alkyl poly(ethylene oxide) Brij-58 but we also investigated the effects of another triblock copolymer with longer hydrophilic segments, Pluronic F127, and of a widely used ionic surfactant, CTAB. The gel films obtained with F127 displayed a lamellar organization of the pores and thus a different distribution of Sr. The microstructure of the annealed films is different from that obtained with films prepared with Brij-58, as they present large and dense star-shaped α-quartz crystals together with a porous α-quartz structure on their surface (see Fig. S9b†). In the case of the films made using CTAB we always observed a phase separation of water droplets within the gel even by decreasing the relative humidity (shown in Fig. S9c†). The same type of phase separation was observed when we used Brij-58 but the films were prepared with an atmosphere with a relative humidity of above 40% (see Fig. S10†).

#### 3.2.2 Withdrawal speed and film thickness

**Fig. 4:**
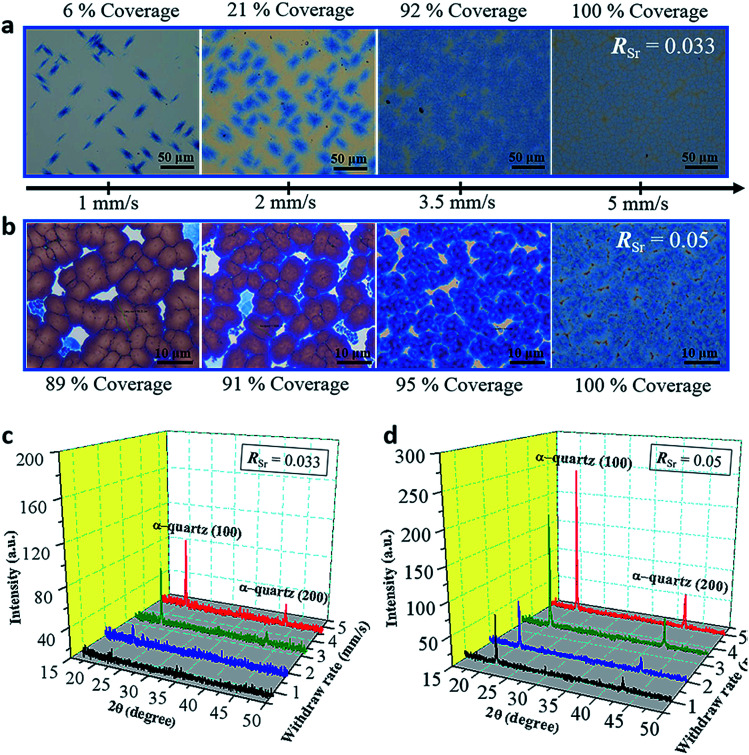
Thickness study for the as-prepared film: (a and b) optical images of films prepared with different withdrawal speeds (indicated in the axis below the images) for *R*_Sr_ = 0.033 (a) and *R*_Sr_ = 0.05 (b). The areal coverages of the porous α-quartz phase for (a) and that of dense α-quartz crystals for (b) are indicated next to the images respectively. The corresponding XRD *θ*–2*θ* measurements are shown in (c) and (d) for the *R*_Sr_ = 0.033 and *R*_Sr_ = 0.05 series, respectively. Those films were prepared in a relative humidity of 40% at 25 °C using Brij-58 as the surfactant. The annealing treatment was at 1000 °C for 300 minutes in an air atmosphere.

The withdrawal speed (*U*_w_) determines the thickness of the gel film (*T*) according to well-established relationships.^31,32^ In our experiments (see Fig. S11†), we obtained films with *T* ranging from 110 nm (with *U*_w_ = 1 mm s^−1^) to 400 nm (for the maximum *U*_w_ = 15 mm s^−1^). For *U*_w_ > 4 mm s^−1^ the thickness of the resulting films is in reasonable agreement with the viscous drag model^31^ which predicts that *T* is proportional to *U*_w_ in log–log scales. In this regime all the films present the same microstructure and the only effect of increasing *U*_w_ is that the resulting film is thicker and we selected *U*_w_ = 5 mm s^−1^ for most of the samples prepared in this research. We studied the influence of *U*_w_ for two Sr concentrations (*R*_Sr_ = 0.033 and *R*_Sr_ = 0.05). Fig. 4a and b show optical microscopy images of these two series of films.

In Fig. 4a the color contrast allows distinguishing the amorphous areas (with lighter tones) from areas covered by α-quartz (in blue tones). In Fig. 4b the films are fully crystallized and the color contrast allows determining the % coverage by dense α-quartz (zones with darker contrast). It can be observed that the crystallized areas increase with higher *U*_w_ for the *R*_Sr_ = 0.033 series. This trend is also reflected in the XRD patterns of the films which display higher intensities of the α-quartz (00*L*) reflections for increasing withdrawal speeds (Fig. 4c). In the case of the *R*_Sr_ = 0.05 series, XRD also shows an increasing intensity for higher *U*_w_, but in this case this is due to the higher surface coverage by large α-quartz crystals with a lower mosaicity (see Fig. S12†). These results reflect the influence of the total amount of Sr in the films which, within a series of constant *R*_Sr_, is proportional to the film thickness. In particular, for *R*_Sr_ = 0.033 we can conclude that fully crystallized films with a 100% surface coverage cannot be obtained for film thicknesses below 200 nm. Analogously, in the case of *R*_Sr_ = 0.05, 200 nm is the minimum thickness to achieve a high coverage (≥95%) of dense α-quartz. These results point to the existence of a threshold in the total amount of Sr that is necessary to obtain homogeneous microstructures of porous (*R*_Sr_ = 0.033) and dense (*R*_Sr_ = 0.05) α-quartz.

### 3.3 Multi-layer film synthesis

**Fig. 5:**
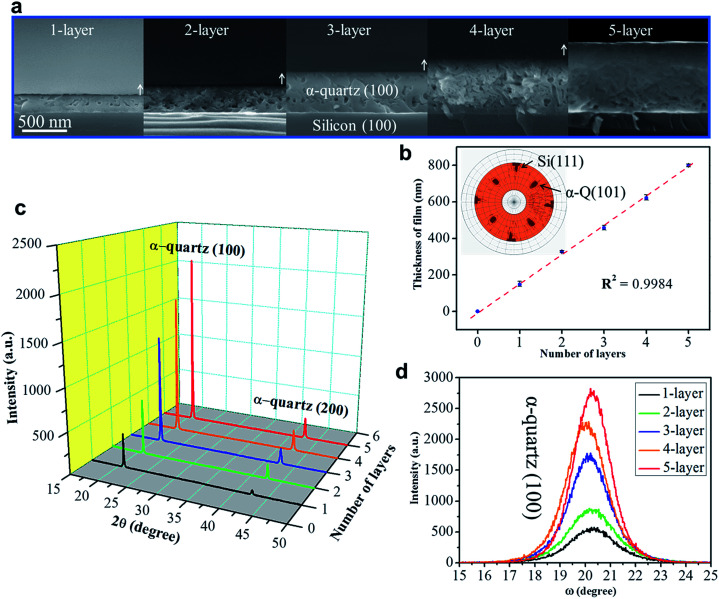
(a) SEM images of the cross-section of the films with different numbers of layers. (b) The linear increase of thickness with the addition of the number of layers; the inserted image is the pole figure for the 5-layer film to show that the epitaxial growth is maintained. (c) XRD *θ*–2*θ* scan showing the linear increase of α-quartz (100) reflections as the number of layers increases. (d) The rocking curves for different multi-layer samples. To prepare these films, all the gel layers were deposited in a relative humidity of 40% at 25 °C with *R*_Sr_ = 0.05 and using Brij-58 as the surfactant. The annealing treatment was at 1000 °C for 300 minutes in an air atmosphere.

To exploit epitaxial α-quartz films in technological applications it is essential to increase the film thicknesses towards the micron range. To this aim we investigated the suitability of a multi-layer deposition approach: the sequential deposition and consolidation of several gel layers before a final annealing treatment to induce the epitaxial α-quartz growth. Fig. 5a presents a series of SEM images corresponding to the cross-sections of films consisting of 1 to 5 layers after the crystallization treatment. One cannot distinguish the interfaces between successive layers and the thickness of the films increases linearly with the number of layers, reaching about 800 nm for the sample consisting of 5 layers (see Fig. 5b). From the XRD measurements of Fig. 5c we can see that all the films present the usual α-quartz (100) out of plane texture and that the intensity of the reflections is proportional to the number of layers of the film. This indicates that the successive layers maintain the crystallinity and crystal orientation with an excellent homogeneity over areas of several cm^2^. Indeed, we have obtained XRD maps on a 5-layer film deposited on a 2-inch Si wafer, showing very slight variations of the α-quartz (100) reflection intensity and the FWHM of the rocking curve over a large area (see Fig. S13†). The intensity of rocking curves of the (100) reflection is also larger for the thicker films and the FWHM is close to 2° for all the films. This shows that the multi-layer approach is not detrimental to the growth of α-quartz films. Further confirmation of this is given by the pole figure of the 5-layer film presented in the inset of Fig. 5b which confirms that the multi-layer synthesis preserves the epitaxial growth of the first layer. It has been argued^33^ that in many inorganic films prepared by chemical solution deposition the maximum achievable thickness is imposed by the cracks due to the lateral tensile stresses which appear during the densification of the layers. With our multi-layer deposition approach we have circumvented this issue by performing a treatment to consolidate the gel layer (450 °C for 10 min in an air atmosphere) after each deposition (see the Experimental section for more details).

**Fig. 6:**
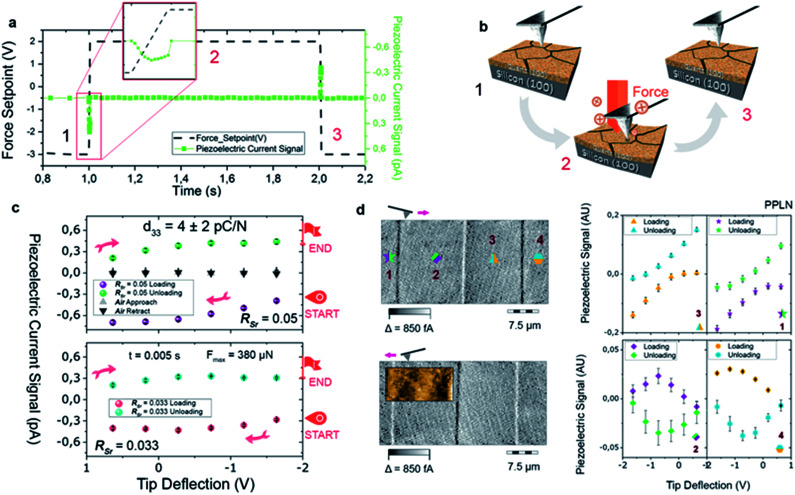
Force and piezoelectric current *vs.* time (s) for different applied forces for a quartz film with *R*_Sr_ = 0.05 (a). The current peaks are seen when DPFM-Si and DPFM-So of a Periodically Poled Lithium Niobate (PPLN) showing the ferroelectric domain configuration of the test sample that is used as a reference for calibration. Scheme of the spectroscopy experiments carried out in which the AFM tip applies a suitable force, within a given time, collecting the charges generated by the direct piezoelectric effect (b). Curves obtained for α-quartz films with *R*_Sr_ = 0.05 and *R*_Sr_ = 0.033 showing their piezoelectric response (c). The graphs were obtained by averaging 4 × 4 matrix volume spectroscopy experiments in an area of 10 microns, in order to depict the homogeneity of the sample. Results of the spectroscopy experiments obtained in four different locations on the PPLN test sample, indicated by the numbers 1 to 4 and the corresponding spots where the curves were recorded (d). Minutes in an air atmosphere.

The availability of epitaxial quartz films with thicknesses approaching a micron made us attempt an unprecedented characterization of the piezoelectric properties by the direct piezoelectric effect. To this aim we employed a novel technique, direct piezoelectric force microscopy (DPFM). In DPFM a conductive AFM tip strains a piezoelectric while the charge that is built up by the mechanical stress induced to the material is being measured.^20^ In order to characterize the piezoelectric response of α-quartz films we performed a step-like force function applied to the material under tests, see Fig. 6a.

The force profile starts with a constant value, while at 1 s the force is increased to a value of 380 μN in a 5 ms time step. Following this step, a constant force is re-settled for an additional 1 s, while at 2 s, an unloading ramp is performed, reducing the applied force. While force is varied, the current channel is recorded simultaneously; current is depicted in green squares in Fig. 6a. Due to the piezoelectric effect, a constant force builds up a constant charge, and hence the recorded current remains zero. However, when the force is varied, through a loading or unloading event, there is an increase or decrease of the charge build up, whereas a constant current can be seen at a constant force rate applied, see Fig. 6b. The system current is calibrated using a known resistance, while the force applied is calibrated using standard procedures available for AFM. To increase homogeneity, the results of our experiments are depicted by averaging 10 curves obtained in 10 different randomized spots of the sample, see Fig. 6c. We were able to obtain the *d*_33_ value of the material by simply dividing the collected current, in pC s^−1^ units, by the force rate applied (0.078 N s^−1^), obtaining the piezoelectric constant in pC N^−1^. Independently of the *R*_Sr_ value, a similar piezoelectric response is measured. As a negative test, we repeated the movement of the *Z*-piezo of the AFM (see black-and-grey triangles of Fig. 6c), obtaining a zero-current response. We performed another test experiment in a ferroelectric Periodically Poled Lithium Niobate (PPLN) sample as a reference (see Fig. 6d). For obtaining the location of ferroelectric domains, DPFM-Si (Signal Input) and DPFM-So (Signal Output) scans are recorded, revealing the expected antiparallel domain configuration.^20^ A similar force ramp consisting of loading and unloading the sample surface with the AFM tip is repeated in each of the domain positions (see Fig. 6d in which the numbers are correlated with right panel graphs). It is seen that the current reverses depending on which domain polarization direction is mechanically stressed. Such behavior is expected for a ferroelectric known sample, leading us to conclude that the recorded current signal arises from the piezoelectric properties inherent to our films. From these measurements we obtained a *d*_33_ of 4 ± 2 pC N^−1^. The reference piezoelectric constants of bulk quartz are *d*_11_ = 2.3 pC N^−1^ and *d*_14_ = −0.7 pC N^−1^.^34^ Since the out of plane orientation of our films is [210] (*i.e.* at 30° with respect to [100]) the obtained piezoelectric constant is in reasonably good agreement with *d*_11_ of bulk quartz (*i.e.* measured along [100]).

### 3.4 Nanostructuring epitaxial quartz films

The enhanced control over the crystallization of epitaxial quartz and the multi-layer deposition approach has enabled us to achieve integrated epitaxial quartz structures on silicon. We demonstrate the feasibility of this integration by combining the dip-coating process to synthesize multi-layers of silica xerogels on silicon (100) substrates, with two complementary patterning techniques. By Laser Interference Lithography we generated silicon masters of highly controlled size, height and pitch of vertical pillars, from which we produced PDMS molds. The molds were used to pattern amorphous silica multi-layers by soft imprint lithography in a fast process which does not require lithographic masks and allows obtaining a precise control of the dimensions over large surfaces (see Fig. S14†).

**Fig. 7:**
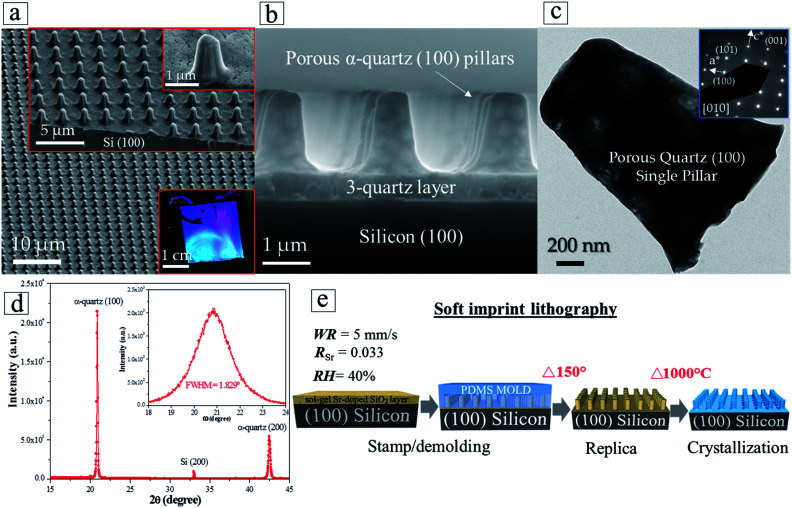
Patterning and crystallization of Sr-doped silica multilayers by soft imprint lithography. (a) FEG-SEM image of the epitaxial quartz columnar pattern. The inset figures show higher magnification FEG-SEM images illustrating the morphology of the quartz columns. The right-down inset corresponds to an optical image of the film which displays the characteristic wavelength reflectance selectivity of a 2-D photonic crystal. (b) Cross-sectional SEM image of a quartz column grown on the Si substrate assisted by the Sr^2+^ catalyst at 1000 °C, 5 hours. (c) Transmission Electron Microscopy image and electron diffraction measurement (inset image) of the α-quartz/Si interface of a single column. (d) *θ*–2*θ* XRD pattern displaying the out of plane texture of the (100) α-quartz crystallographic phase in the patterned film. The inset shows a rocking curve indicating that the mosaicity value of quartz is not affected by the lithography process. (e) Schematics summarizing the key steps that were applied to produce patterned epitaxial quartz films by soft imprint lithography.

To illustrate the possibilities of this approach we prepared cm-scale areas of epitaxial quartz patterned with pillars on a multi-deposition silica film with four layers. Fig. 7a and b show different magnification SEM-FEG images of a patterned quartz film displaying pillars of 1 μm diameter and 2 μm in height. The optical image of the whole patterned film presented in the lower-right inset of Fig. 7a displays the characteristic wavelength-selective reflectance of 2D-photonic crystals, indicating the good quality of the patterning over an area of several square centimeters. The electron diffraction of a single column (Fig. 7c) and X-ray diffraction *θ*–2*θ* and *ω* scans of the film (Fig. 7d) show that, as in non-patterned films, it consists of epitaxial quartz with Si (100)//α-quartz (100) and Si (010)//α-quartz (010). It is noteworthy that the epitaxial growth has been transferred to the pillars. With regard to these, the multi-layer deposition approach is highly relevant because the first three silica layers act as crystallization seeds and facilitate the adhesion of the fourth layer to faultlessly replicate the columnar shapes when it is imprinted by the PDMS mold. The process to prepare patterned epitaxial quartz (Fig. 7e) involves a first step of imprinting a Sr-doped amorphous SiO_2_ gel followed by a crystallization treatment at high temperature. Comparing the patterned films before (Fig. S13b and c†) and after (Fig. 7b) crystallization one can see that the shape of the patterned structures is highly preserved during the whole process. Indeed, no cracks or other defects are generated upon crystallization, in spite of the substantial thermal stresses that can be developed for pillar dimensions exceeding 1 μm and temperature differences in the order of 1000 °C. If the multi-layer deposition approach allows preparing thicker epitaxial films above 500 nm (see Section 3.3), we have shown that in combination with soft imprint lithography it is possible to obtain features with vertical dimensions in the micron range.

## 4. Conclusions

We have shown that the total amount of devitrifying agent in mesoporous silica films prepared by dip-coating determines the microstructure of epitaxial α-quartz after thermal devitrification. In our study the quantity of Sr^2+^ was controlled by both the ratio of Sr^2+^ to SiO_2_ (*R*_Sr_) and the withdrawal speed (*U*_w_) during dip-coating. In particular, with *U*_w_ = 5 mm s^−1^, for *R*_Sr_ = 0.033 one obtains porous α-quartz films with randomly distributed macropores of sizes below 300 nm. In contrast for *R*_Sr_ ≥ 0.05 the microstructure is dominated by dense α-quartz crystals of several microns in size. Our study has also established the importance of controlling the relative humidity and the nature of the surfactant. We achieved the targeted microstructures (porous or dense α-quartz epitaxial films) with a relative humidity of 40% and using the non-ionic surfactant Brij-58. The possibility of controlling these contrasting microstructures by simply adjusting the content of devitrifying agent paves the way for further developments exploiting the potentialities of α-quartz films on Si (100) for specific applications. Namely, the films displaying larger dense α-quartz structures are best placed for the development of optical devices as the size of the crystals is sufficiently large to enable the fabrication of photonic structures by lithographic techniques. In contrast, the porous films are well-suited for microgravimetric sensing applications as the pores can be further functionalized to provide an enhanced selectivity towards analytes and increase the selectivity and detection limits. An additional step forward towards these sensing applications is the suitability of the multi-layer synthesis to extend the thickness of the α-quartz films prepared by dip-coating. By combining this method with soft imprint lithography, we have demonstrated that one can obtain patterned epitaxial quartz films. These findings open up new horizons for integration of quartz-based nanostructures on silicon from which many applications relying on electromechanics, optics and optomechanics would benefit.

## Conflicts of interest

There are no conflicts to declare.

## Supplementary Material

NA-001-C9NA00388F-s001
