# Supplementary material for: Tailoring the crystal growth of quartz on silicon for patterning epitaxial piezoelectric films
Source: Nanoscale Adv. 2019 Aug 29;1(9):3741–52. doi: 10.1039/c9na00388f (PMC9419616; doi:10.1039/c9na00388f)
Supplement: NA-001-C9NA00388F-s001 [file NA-001-C9NA00388F-s001.pdf]

Electronic Supplementary Material (ESI) for Nanoscale Advances.
This journal is © The Royal Society of Chemistry 2019

Supplementary Information

Tailoring the crystal growth of quartz on silicon for patterning piezoelectric films

Qianzhe Zhang[a,b], David Sánchez-Fuentes [b], Andrés Gómez [a], Jaume Gàzquez [a], Adrián Carretero-Genevrier [b] and Martí Gich [a].

[a] *Institut de Ciència de Materials de Barcelona ICMAB, Consejo Superior de Investigaciones Científicas CSIC, Campus UAB, 08193 Bellaterra, Catalonia, Spain.*
[b] *Institut d'Électronique et des Systèmes (IES), UMR 5214, CNRS - Université de Montpellier, 860 rue Saint Priest, 34095 Montpellier, France.*

Table S1. Correspondence between the molarity of the $SrCl_2$ aqueous solution and the Sr to SiO_2 molar ratio in the final solution used in the dip-coating process.

**Concentration of the $SrCl_2$ aqueous solution (Solution B)**	**Sr/Si molar ratio in the final solution (Solution C)**
0.4 M	0.02
0.5 M	0.025
0.6 M	0.03
0.65 M	0.0325 (shown as 0.033 in paper)
0.7 M	0.035
0.75 M	0.0375 (shown as 0.038 in paper)
1 M	0.05
1.5 M	0.075
2 M	0.1

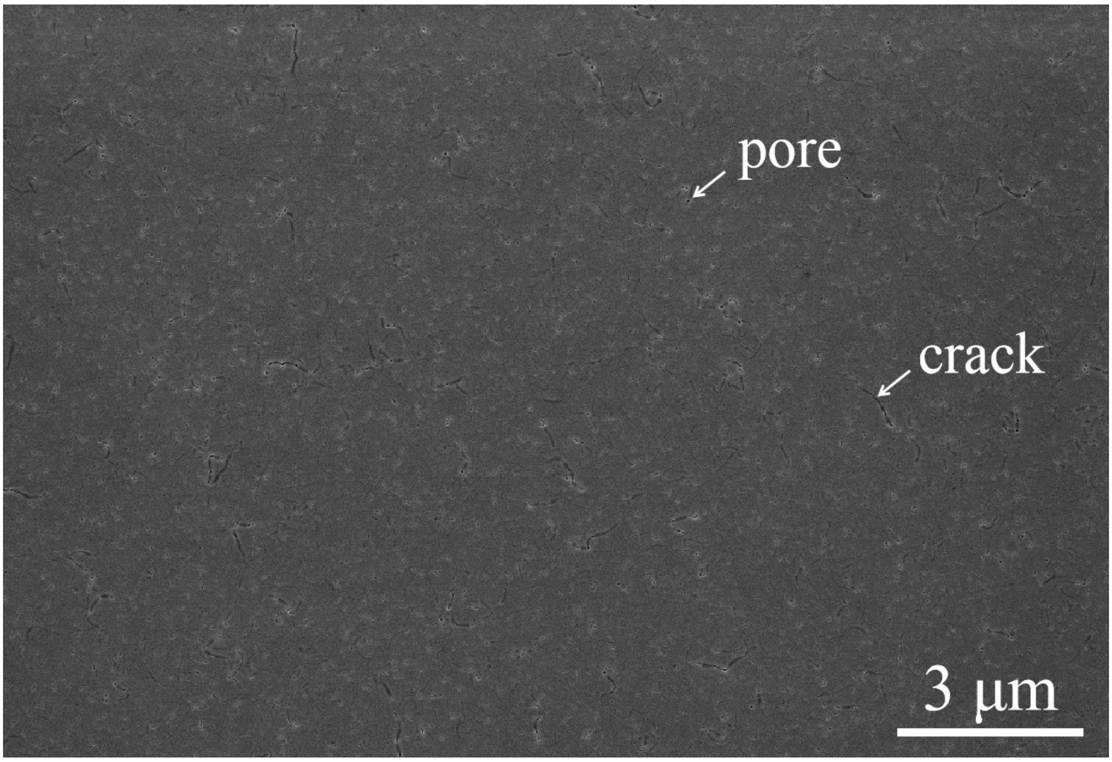

Figure S1. SEM image in secondary electrons mode of a film without Sr, showing the presence of cracks and pores.

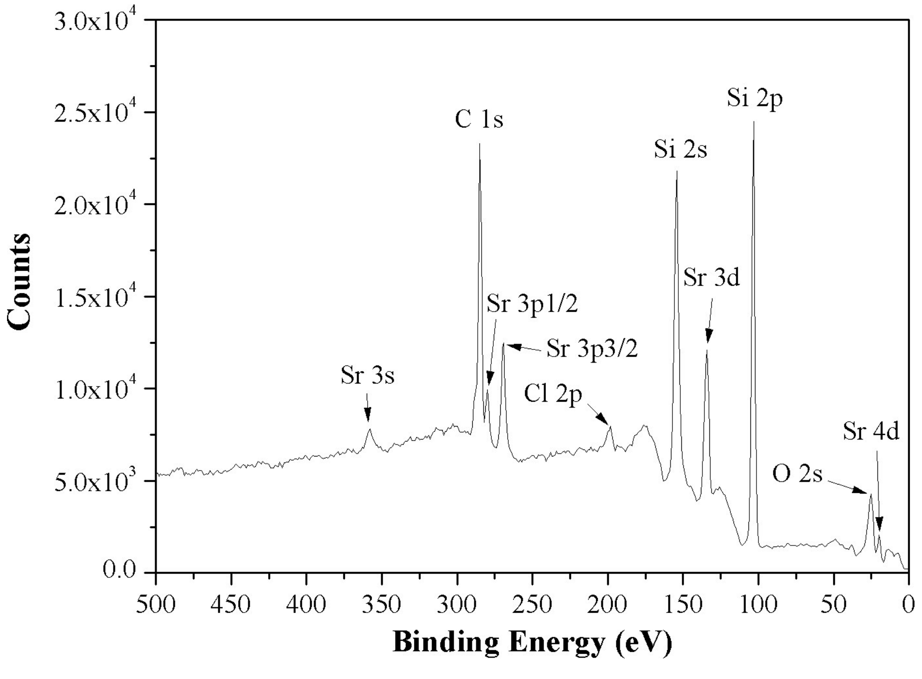

Figure S2. XPS overview of $\boldsymbol{R}_{Sr}$ = 0.05 film to show the present of Sr components

inside film.

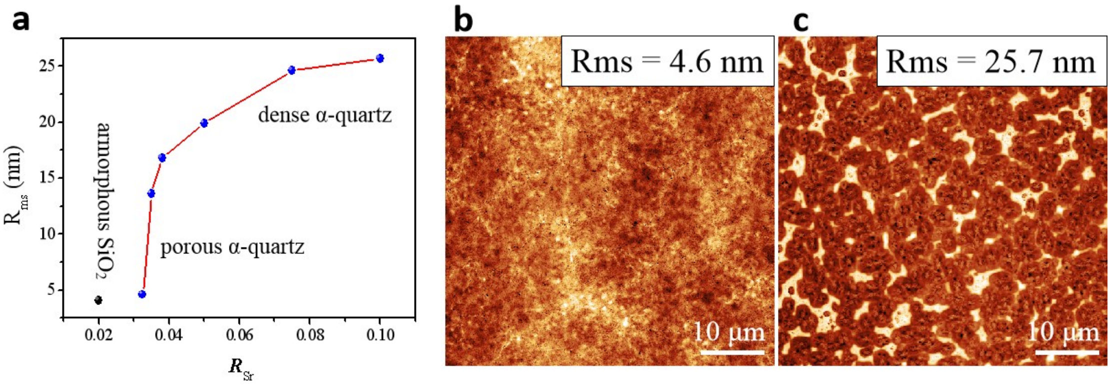

Figure S3. Topography study for different $\boldsymbol{R}_{Sr}$: **(a)** The relationship between with $\boldsymbol{R}_{Sr}$ and film roughness (R_{ms}) **(b)** Topographic AFM image of the porous film ($\boldsymbol{R}_{Sr}$=0.033) with the lowest R_{ms}, **(c)** Topographic AFM image of a dense film (with $\boldsymbol{R}_{Sr}$=0.1) displaying the largest R_{ms}.

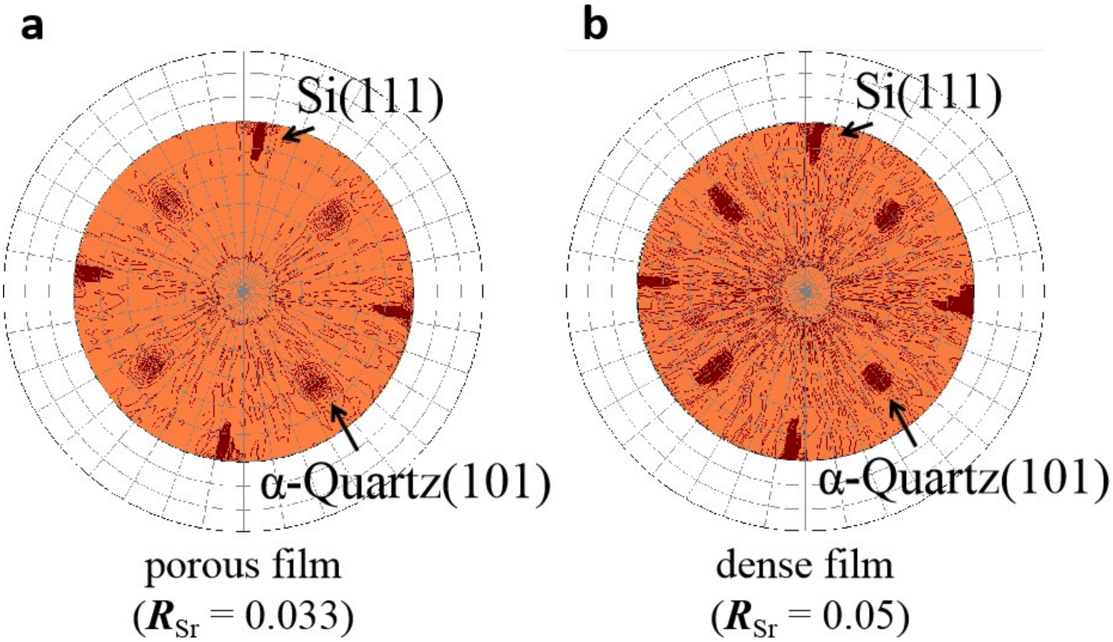

Figure S4. Pole figure of (101) α-quartz for two characteristic microstructures: **(a)** porous film ($\boldsymbol{R}_{Sr}$ = 0.033), **(b)** dense film ($\boldsymbol{R}_{Sr}$ = 0.05).

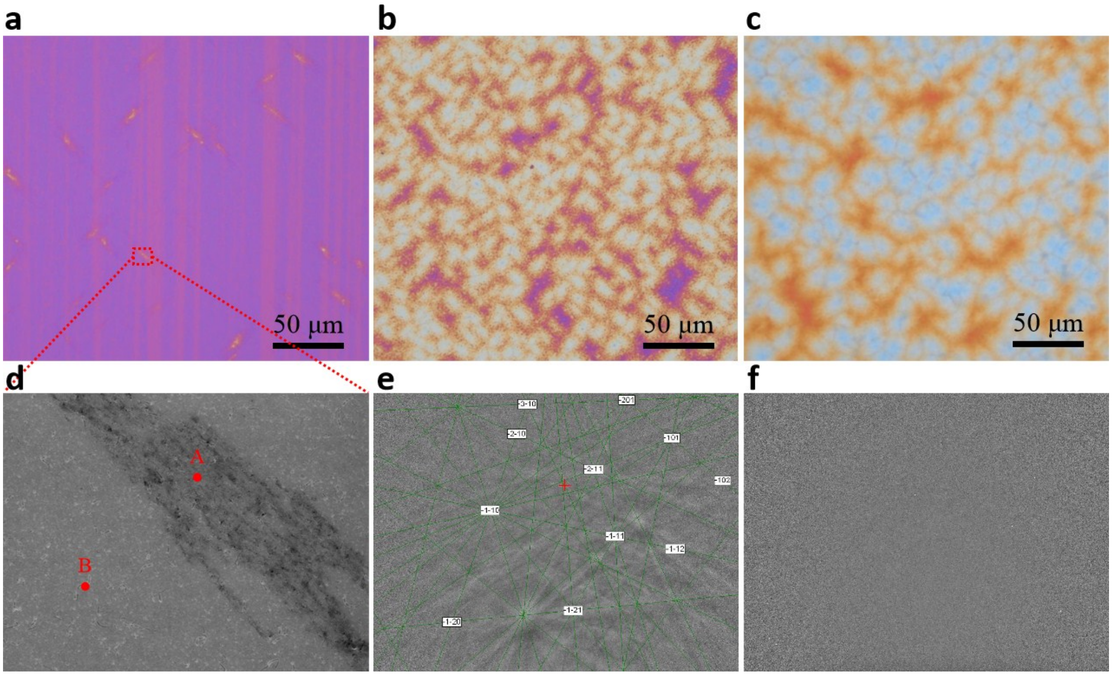

Figure S5. Optical images of partially crystallized films when $\boldsymbol{R}_{\mathrm{Sr}}$ is **(a)** 0.02, **(b)** 0.025, **(c)** 0.03. In these images, only the light color areas are crystallized to α-quartz phase, the remaining violet and brown areas are still amorphous. **(d)** is the SEM image of one selected zone of $\boldsymbol{R}_{\mathrm{Sr}}$ = 0.02 condition film which containing crystalline structure (dark area) and amorphous surface (light area) for EBSD test. **(e)** is the EBSD result of point A in **(d)** with α-quartz phase signals. **(f)** is the EBSD result of point B in **(d)** without any signal.

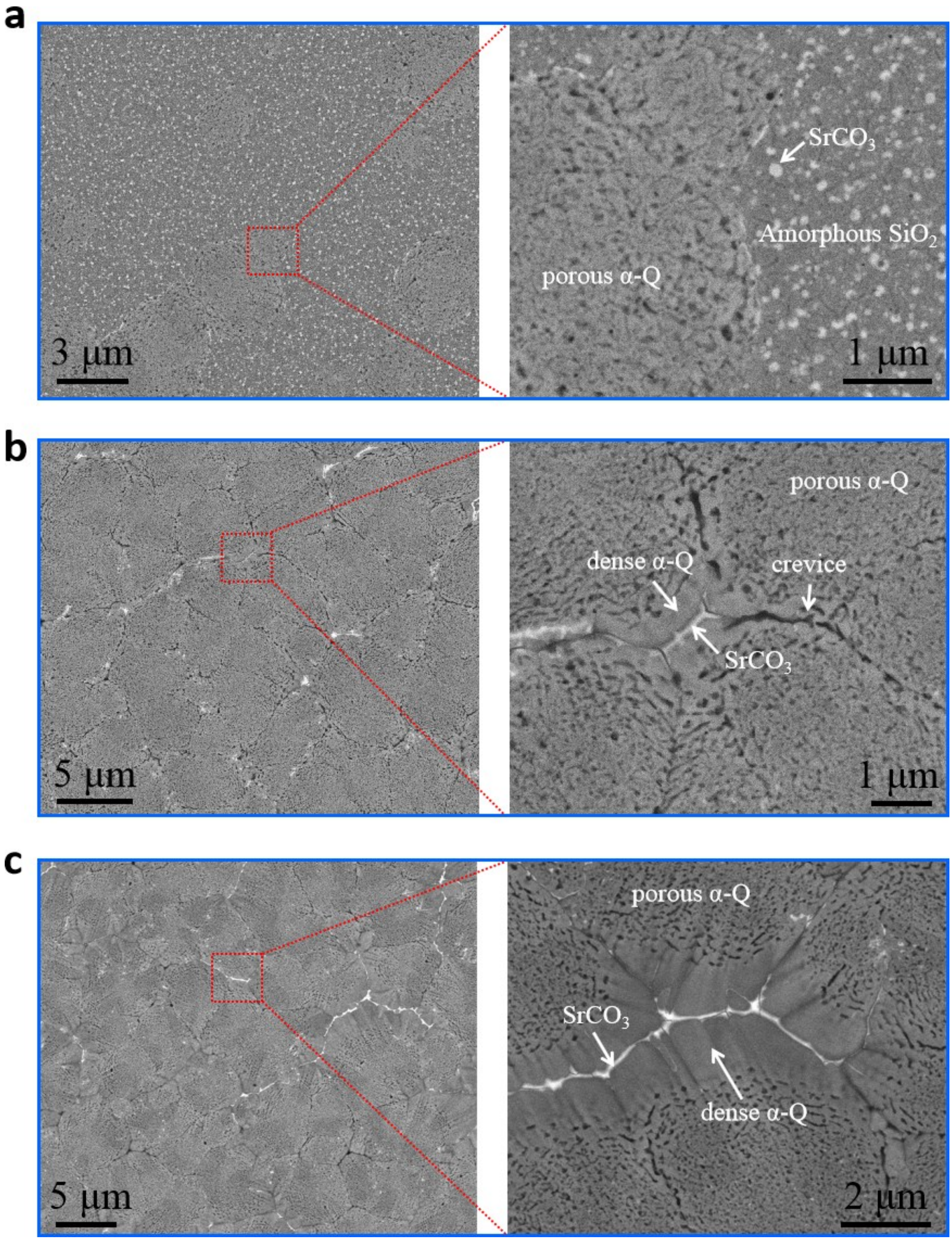

Figure S6. SEM images in secondary electrons mode of $\boldsymbol{R}_{\mathrm{Sr}}$ = 0.035 films crystallized with different times at 1000ºC: **(a)** 40 min. **(b)** 50 min. **(c)** 60 min.

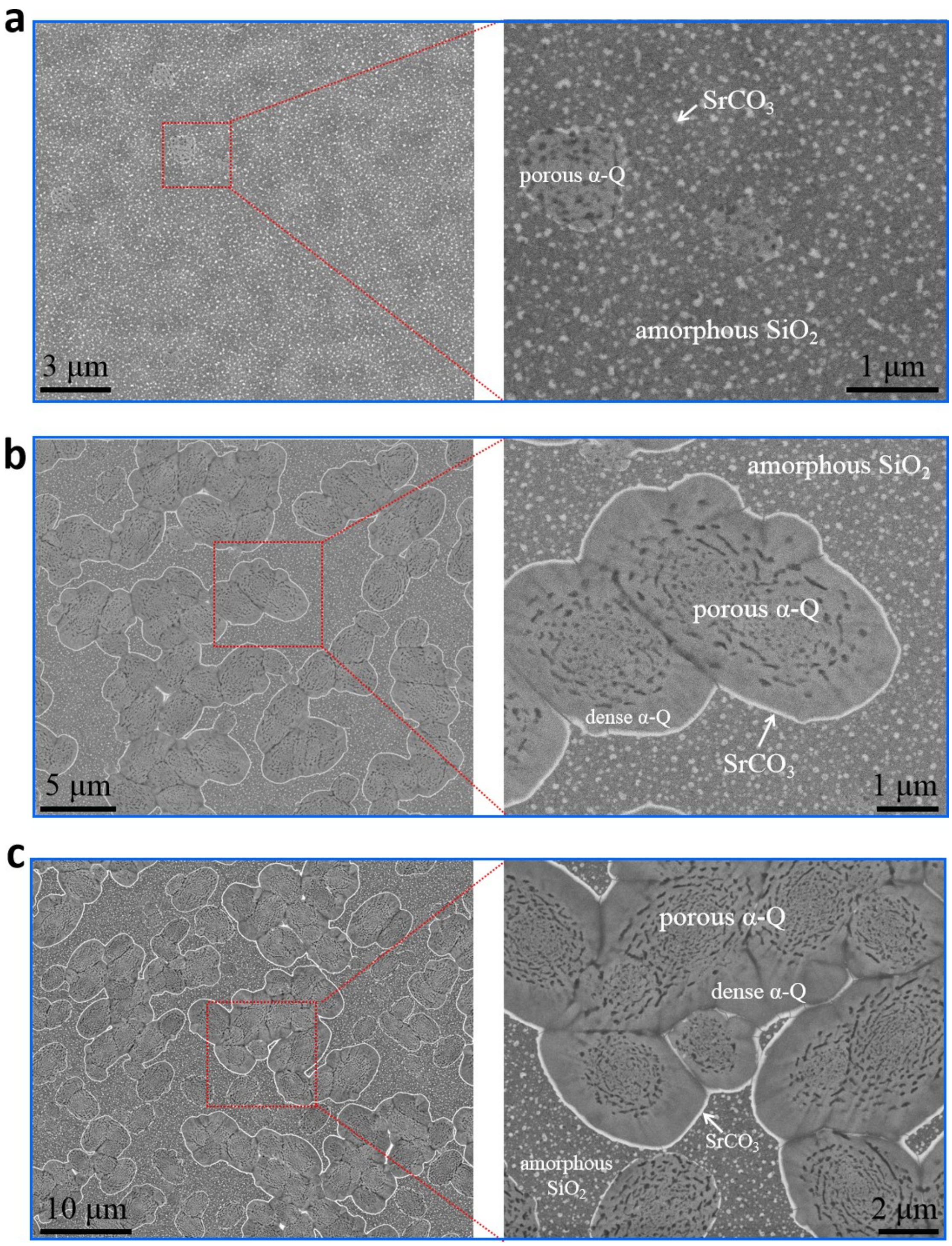

Figure S7. SEM images in secondary electrons mode of $\boldsymbol{R}_{Sr}$ = 0.05 films crystallized with different annealing times at 1000ºC: **(a)** 20 min. **(b)** 30 min. **(c)** 60 min.

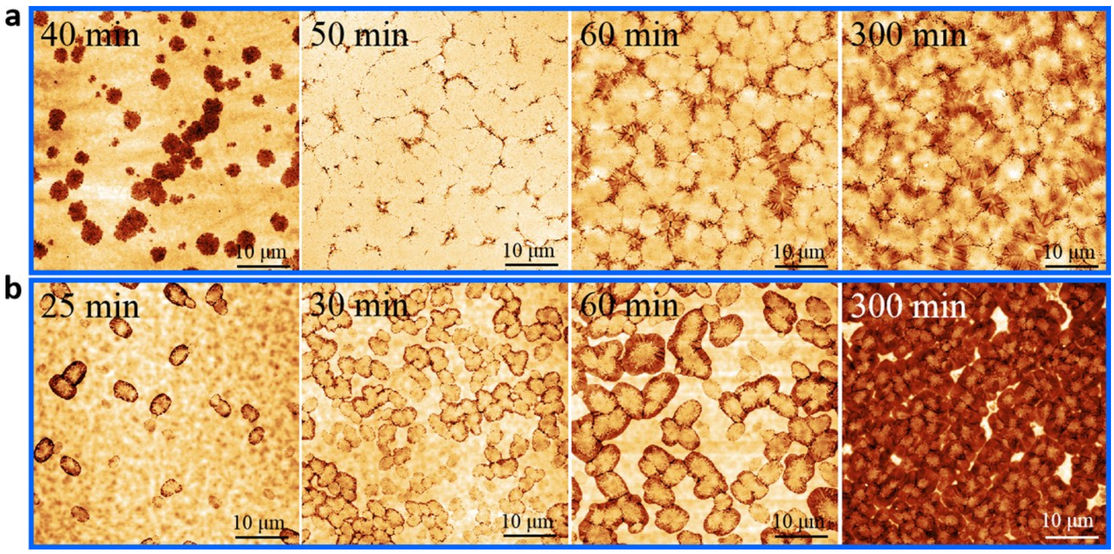

Figure S8. Topographic AFM images showing the influence of annealing time at 1000ºC for **(a)** porous microstructure (obtained with $\boldsymbol{R}_{\mathrm{Sr}}$ = 0.035), **(b)** dense microstructrure (obtained with $\boldsymbol{R}_{\mathrm{Sr}}$ = 0.05).

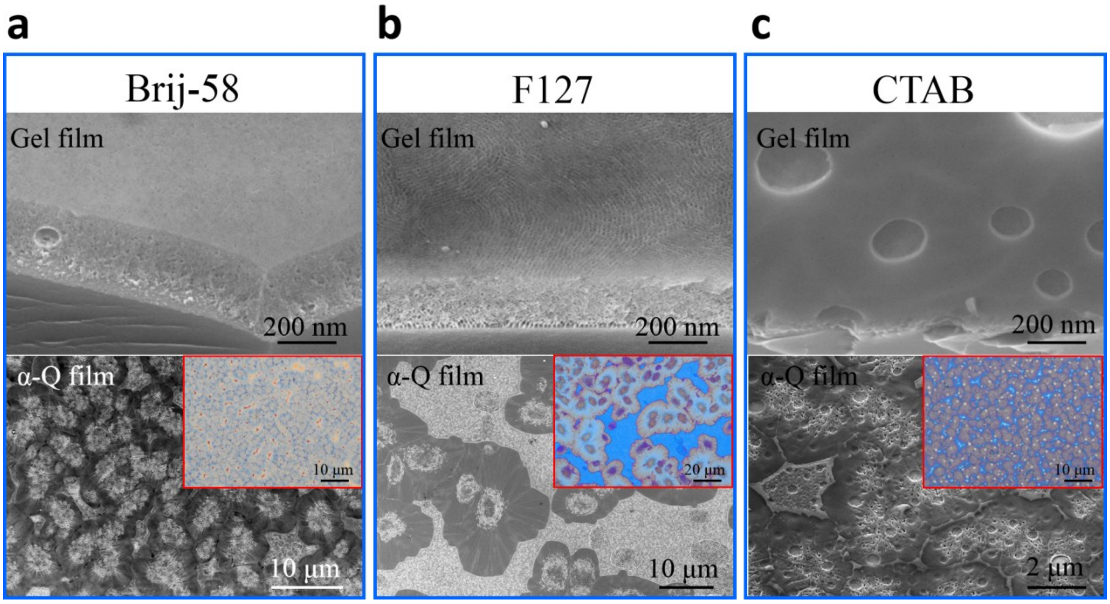

Figure S9. SEM pictures of gel films with $\boldsymbol{R}_{\mathrm{Sr}}$ = 0.05 (upper panel) and the corresponding annealed films for 300 min at 1000ºC (lower panel) with optical images in as an inset using three different three surfactants: **(a)** Brij-58, **(b)** F127, **(c)** CTAB.

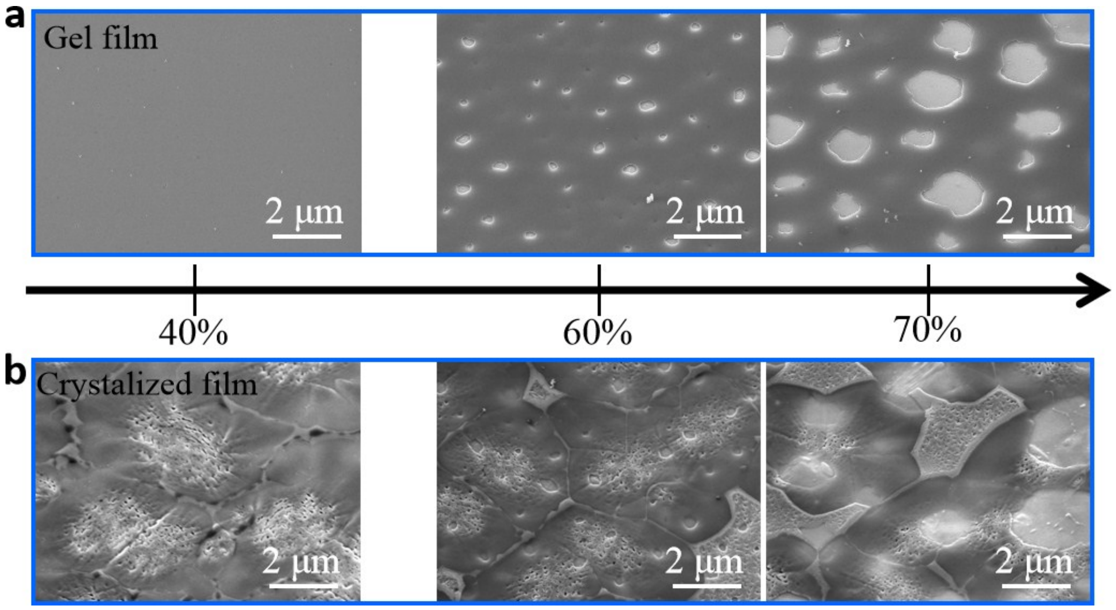

Figure S10. SEM pictures of films with $\boldsymbol{R}_{\mathrm{Sr}}$ = 0.05 prepared at three different relative humilities indicated in the axis next to the images: **(a)** gel films **(b)** after annealing for 300 min at 1000ºC.

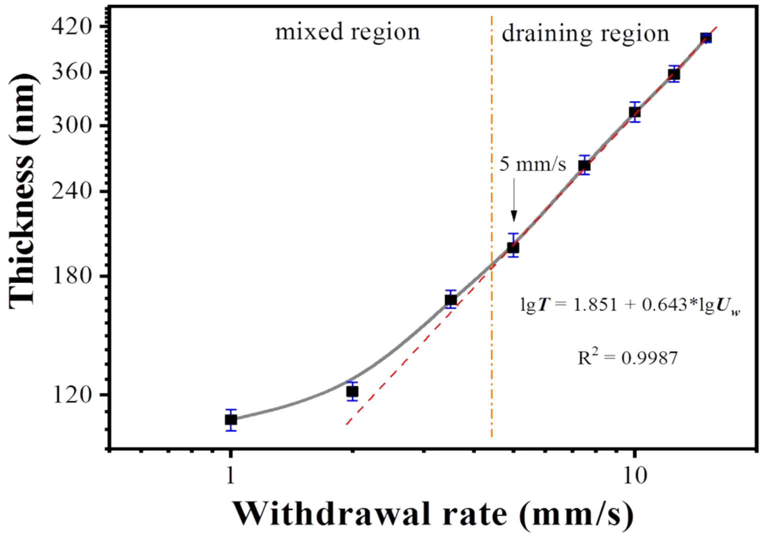

Figure S11. Thickness measurement of gel films with $\boldsymbol{R}_{\mathrm{Sr}}$ = 0.05 and prepared using different withdrawal speeds showing the increasing gel film thickness with $\boldsymbol{U}_{\mathrm{w}}$: and the different regimes: the viscous drag regime for $\boldsymbol{U}_{\mathrm{w}}$ > 4 mm/s and the mixed regime, for $\boldsymbol{U}_{\mathrm{w}}$ < 4 mm/s.

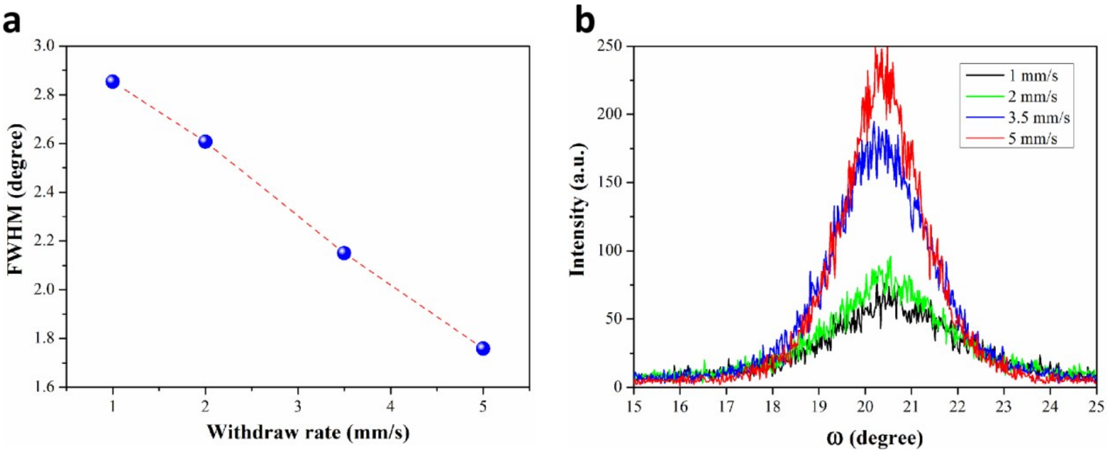

Figure S12. The FWHM analysis of R_{Sr} = 0.05 films made by different withdraw rates is shown as **(a)**. Meanwhile, the raw data of rocking curves at α-quartz (100) peak are shown as **(b)**.

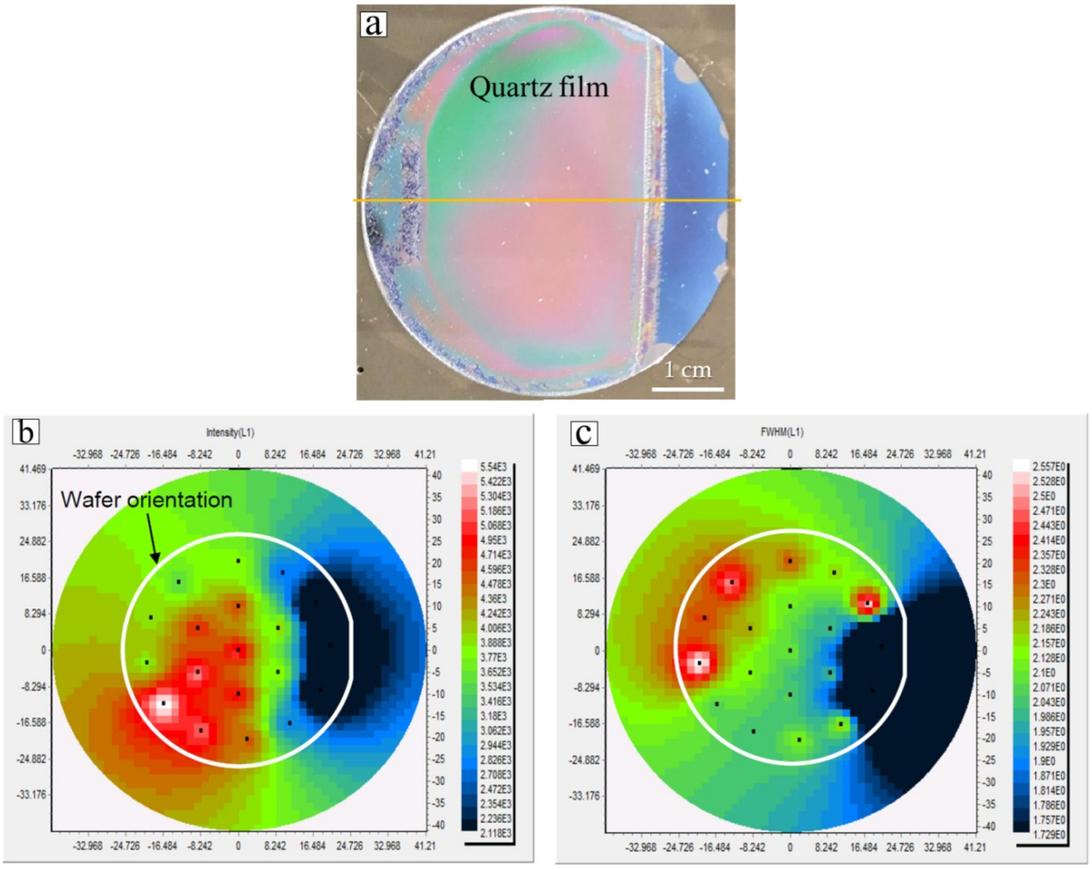

Figure S13. A 2 inch epitaxial quartz thin film on silicon substrate fabricated by 5 multilayer deposition, reaching a final thickness of 800 nm **(a)**. Heat map of peak intensity of (100) quartz reflection **(b)**. Heat map of evaluated peak width (FWHM) of rocking curves around quartz (100) reflection **(c)**.

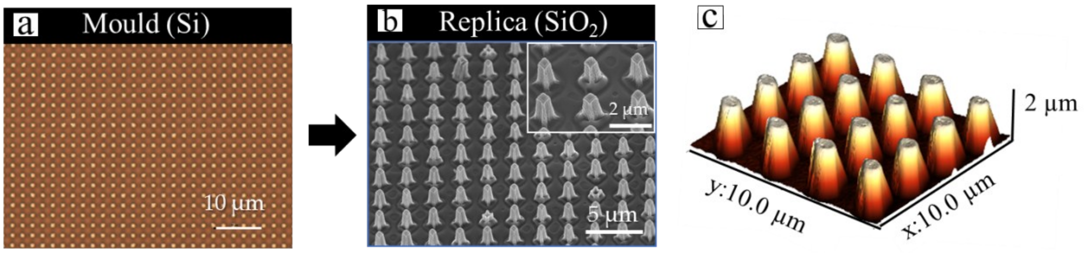

Figure S14. (a) Optical image of Si (100) masters used along this work obtained by using LIL lithography**. (b)** FEG-SEM image of printed Sr-doped silica columns with a diameter of 1 µm and height of 2 µm on silicon. The inset pictures shows FEG-SEM images at higher magnification of pillars. **(c)** 3D AFM images of the nanostructured silica films prepared by NIL lithography.
